# Pyorrhœa Alveolaris

**Published:** 1894-01

**Authors:** F. T. Van Woert


### PYORRHŒA ALVEOLARIS.[1]

BY DR. F. T. VAN WOERT.

THERE is a possibility that I may get myself into some difficulty before the evening is over, but shall not regret in the least having done so if I can provoke a discussion which will throw some light on the subject I have to present.

Much has been said and written relative to so-called "Riggs's disease," or pyorrhœa alveolaris. In the past seventeen years many theories have been advanced as to the conditions of both hard and soft tissue, and various treatments have been recommended; still, thousands of teeth are lost every year from a lack of knowledge as to what the disease really is, of its origin, and how to treat it.

Like many of you, I have felt very much discouraged in the efforts to relieve my patients of this dreaded difficulty, and after a vast amount of wasted labor and energy, to say nothing of the great anxiety suffered, I decided about two years ago that I must abandon all hope of success in combating the trouble, unless I could find a different theory of the disease and consequently a new treatment. It was my intention to place this matter before you in a way that would leave as little doubt in your mind of the accuracy of my conviction as in my own. Please do not misconstrue this as meaning that I claim to have solved the mystery, but simply credit me with sincerity in my conversion to the statements made, and either join me in the new faith or show me the error of my ways, that I may not labor under a delusion.

I regret exceedingly circumstances have made it necessary to present this paper in this crude form: in the line of my investigation it has been a very difficult matter to secure specimens that I considered of sufficient value to prove the theory. My labors having been in the dissecting-room and at autopsies; such cases as were of value to me were held by students who would not give them up, and I could not mutilate the bodies upon which autopsies were held without disfiguring the face to make it noticeable to the friends

---

[1] Read before the New York Odontological Society, October 17, 1893.

of the deceased. I hope, however, and expect to get sections to prove all that I claim, and which will be considered worthy of space in the collections owned by this Society.

Such cases as I examined in the dissecting-room showed the loss of hard and soft tissues, calcareous deposits, and all the symptoms, other than the flow of pus, that the disease had been present. In four cases I had the good fortune, through the courtesy of friends in the medical profession, to be called in consultation upon persons suffering with genuine so-called pyorrhœa alveolaris and other incurable diseases, and subsequently was present at the autopsies and made careful examinations in each case without finding the least trace of necrosis. Could I have presented sections from these I am confident that you would believe as I do.

To distinguish this form of carious bone in the mouth of the living I contend is almost an impossibility; the alveoli, being a calsenious or spongy tissue, responds to the sense of touch to all the symptoms of a marked necrosis, and an exposure of the parts in other than the cadaver is out of the question; hence to diagnose in the mouths of patients as they present themselves to us is not practical unless this theory of necrosis be discarded.

If this is true, the operation recommended by Dr. Riggs and very generally practised is productive of more harm than good. Perhaps my manipulation in the performance of this operation has been imperfect, which would account for failure; but the fact that partial success has crowned my efforts in the avoidance of this treatment and the substituting of one almost the reverse, leads me to believe that I am not far wrong in my conclusions. I am satisfied that many members of our profession have had doubts as to the existence of necrosis in this disease. Two or three writers have said that there were but few cases where it was present, and I can but feel if they would look into the matter again, pursuing the course I speak of, they will proclaim, as I have, it never is. And I claim, as well, that there is but one form of this disease; that there are a variety of difficulties which may cause the trouble, I fully appreciate, but I do not believe that this is part of it. It may result in the same proportion that pneumonia or phthisis would follow a cold if not cared for in time. The great mistake has been that all the ailments of these parts have been considered a type of this difficulty, and little or no effort to so name them that a true diagnosis could be made.

I cannot better express myself than to quote from Dr. G. V. Black, vol. i. p. 953, in the "American System of Dentistry." And while his article was written some years ago, still our position is practically the same to-day in this respect. He says,—

"This group of diseases has generally been passed over without very accurate description. They have universally been grouped together under one name. This name has varied with the different writers to such an extent as to have almost as many names as authors." And he felt that the time had come when the old names should be dropped and others introduced that are more in harmony with what is now known of the pathological condition present in each case.

This he acknowledges to be a difficult task, but absolutely necessary to accuracy. "It cannot be expected that the profession at large will have definite ideas of these diseases until we have definite descriptions under definite names. The term gingivitis I limit to those inflammations of the gingivæ that occur from constitutional causes, or the lighter forms of inflammation from soft deposits on the teeth. It may be argued, and justly, that all of the diseases of this class begin with an inflammation of the gingivæ; but when another factor has entered into the case, probably in its inception, it is proper that that factor should be expressed; hence the term calcic inflammation, indicating directly the nature of the cause that perpetuates the disease. This is seen in two forms, serumal and salivary calculus; but as these relate to the origin of the calculus that induces the inflammation rather than to the character of the inflammation itself, and as the two are often blended together in the same case, it has hardly seemed to call for a separate name. In the term phagedenic pericementitis I have again expressed the most prominent factor of the affection that is at the present time definitely determined,—its destructive character. It is true that the calcic form is destructive, but not to the same extent. This disease is, in my opinion, caused by a specific form of micro-organism, but this has not been determined with sufficient accuracy to justify a name expressive of that conception.

"The names heretofore in use have been applied to the entire group of diseases, and among them no distinctions have been made. The most of them are now no longer used. The term pyorrhœa alveolaris expresses one fact common to all of these forms after they have made considerable progress, including alveolar abscess as well,—a flow of pus from the alveolus. It must be seen by all that when we come to a classification of these affections this term loses all distinctiveness and cannot be of use. Possibly this name might be retained as expressive of the whole group of diseases in which there is a flow of pus from the alveolus, but this could not be of much value; especially is it objectionable after the use that has been made of it in the past."

This is about what I desire to say, other than that he does not deny the presence of necrosis. The disease which proves so fatal is one, I believe, confined to the peridental membrane, brought about by any of the other diseases to which the mouth is subject, and very often by inoculation in the use of instruments imperfectly sterilized, or rubber dam which has been in contact with the exudation from the pockets where the disease was in progress. I feel convinced there are many modes of conveying it from one mouth to another. No doubt many of you will not believe this, but I apprehend the day is not far distant when you will concede it as you have been forced to accept many other theories that seemed quite as ridiculous. My convictions are based upon practical experiment and careful research, and are not a mere fancy, and it will take more than a simple opinion to change my own views.

The loss of the alveoli is, I believe, due to a process of absorption such as takes place after the extraction of a tooth, and is an effort on the part of nature to dispose of that which has ceased to be of service. The weeping of serum from the diseased membrane calcifying upon the roots destroys the union of bone and tooth; and thus the function of the bone at that point becomes a thing of the past, hence its absorption.

In my method of treatment I begin with the first principles of modern surgery,—that of thorough sterilization of everything connected with the operation and the free use of antiseptics during it. First thoroughly free the teeth and surrounding parts of all food secretions, etc., without lacerating the soft tissues or getting the débris into the pockets; this is done with wheel-brushes on the engine, scalers, strips of orange-wood, and the atomizer, using a three-per-cent. solution of pyrozone in the latter, and frequent washing of the mouth with a solution of lysol; then, with a Koch's hypodermic syringe, the points of which have been ground smooth so that they will not puncture the gums, I wash the pockets with a five-per-cent. solution of pyrozone and follow by swabbing, so to speak, with the twenty-five-per-cent. solution of the same drug. This is accomplished by winding cotton on the points of orange-wood sticks shaped for that purpose, care to be taken in cleansing and sterilizing the hands between each operation. I keep two large finger-bowls on the bracket, filled with antiseptic solutions for freeing the mouth-mirror, instruments, etc., and a good supply of napkins to prevent contamination from contact with any of the removed substances. I next remove as much as possible of the calcareous deposits without encroaching upon, or injury to, the peridental membrane, avoiding the alveolus; then I shape orange-wood like chisel scalers, sear the surface in the flame of a spirit-lamp or Bunsen burner, and with this I apply a saturated solution of trichloracetic acid, by dipping the points in the acid, and use them as in the act of scaling with steel instruments. The remaining calcareous matter can be softened so that all can be removed. Care should be taken to keep the pockets as free from saliva as possible during this last operation, which, when completed, I am satisfied of the removal of all the deposits; I wash again with the three-per-cent. pyrozone, applied with the syringe and atomizer.

It is rare that I operate on more than one-half of either the superior or inferior at one sitting, and allowing from six to ten days between each, and where there has been waste enough of the tissues to cause the teeth to be loose after healing I bridge them as recommended by Dr. Rhein. Many thanks to him. And for the great assistance of trichloracetic acid, I am under obligations to Professor Peirce. I am very much indebted to Dr. Allport for a part of the instruments suitable for the operation.

In conclusion, do not misunderstand this as meaning that I always cure this disease; in fact, I fear I never do, and to the date of this I know of no other treatment that can claim more, notwithstanding the positive declarations by some of the radicals in the old belief. All that I claim is a simple means of controlling the disease sufficient to keep many teeth in the mouth in a comparatively healthy condition long after they would otherwise be lost.

